# Molecular Imbalances Between Striosome and Matrix Compartments Characterize the Pathogenesis and Pathophysiology of Huntington’s Disease Model Mouse

**DOI:** 10.3390/ijms26178573

**Published:** 2025-09-03

**Authors:** Ryoma Morigaki, Tomoko Yoshida, Joji Fujikawa, Jill R. Crittenden, Ann M. Graybiel

**Affiliations:** 1 McGovern Institute for Brain Research, Massachusetts Institute of Technology, Cambridge, MA 02139, USA; morigaki.riyoma.1@tokushima-u.ac.jp (R.M.); yoshidat@mit.edu (T.Y.); jrc@mit.edu (J.R.C.); 2 Department of Brain and Cognitive Sciences, Massachusetts Institute of Technology, Cambridge, MA 02139, USA; 3 Department of Advanced Brain Research, Graduate School of Biomedical Sciences, Tokushima University, Tokushima 770-8503, Japan; joji@tokushima-u.ac.jp; 4 Department of Neurosurgery, Graduate School of Biomedical Sciences, Tokushima University, Tokushima 770-8503, Japan; 5 Parkinson’s Disease and Dystonia Research Center, Tokushima University Hospital, Tokushima 770-8503, Japan

**Keywords:** PDE10A, olfactory type G-protein alpha subunit, striosome, neostriatum, movement disorders, dopamine, chorea, Huntington’s disease, dystonia

## Abstract

The pathogenesis and pathophysiology of Huntington’s disease (HD) are still incompletely understood, despite the remarkable advances in identifying the molecular effects of the *Htt* mutation in this disease. Clinical positron emission tomography studies suggest that phosphodiesterase 10A (PDE10A) declines earlier than dopamine D1 and D2 receptors in HD, indicating that it might serve as a key molecular marker in understanding disease mechanisms. In movement disorders, mutations in the genes encoding PDE10A and G-protein α subunit (Gα_olf_), both critical cAMP regulators in striatal spiny projection neurons, have been linked to chorea and dystonia. These observations highlight the potential importance of striatal cyclic AMP (cAMP) signaling in these disorders, but how such dysfunction could come is unknown. Here, we suggest that a key to understanding signaling dysfunction might be to evaluate these messenger systems in light of the circuit-level compartmental organization of the caudoputamen, in which there is particular vulnerability of the striosome compartment in HD. We developed machine learning algorithms to define with high precision and reproducibility the borders of striosomes in the brains of Q175 knock-in (Q175KI) HD mice from 3–12 months of age. We demonstrate that the expression of multiple molecules, including Gα_olf_, PDE10A, dopamine D1 and D2 receptors, and adenosine A2A receptors, is significantly reduced in the striosomes of Q175KI mice as compared to wildtype controls, across 3, 6, and 12 months of age. By contrast, mu-opioid receptor (MOR1) expression is uniquely upregulated, suggesting a compartment-specific and age-dependent shift in molecular profiles in the Q175KI HD mouse model caudoputamen. These differential changes may serve as a useful platform to determine factors underlying the greater vulnerability of striatal projection neurons in the striosomes than in the matrix in HD.

## 1. Introduction

The differential neurodegeneration of the striosome and matrix compartments has been found in postmortem neuropathologic studies of the striatum in patients with Huntington’s disease (HD) [1,2,3,4,5,6,7,8,9,10,11,12]. Further, abnormalities in gene regulation in the striosome–matrix axis or organization have also been found by single-nucleus RNA sequencing (snRNA-seq), accompanying the well-known decline in the striatal projection neurons (SPNs) expressing dopamine D2 (Drd2) receptors [13]. Examination of a Grade 1 case has demonstrated that striosomal SPNs are the first to degenerate in HD [12], and an snRNA-seq study has suggested that the most vulnerable SPN cell type is striosomal SPNs expressing Drd2 receptors [13]. It has further been suggested that this early loss relative to matrix SPNs reflects a striosome–matrix imbalance aligned with the emergence of premanifest symptoms, particularly mood and cognitive disturbance that typically precede overt motor signs. The precise process of molecular alterations in these compartments that cause neuronal death and aberrant brain function in patients with HD is largely unknown. Hindering these studies is the small diameter of most of the striosome components (~0.5–1 mm in the human) and their labyrinthine distribution within the much larger matrix compartment [14]. These constraints have so far made imaging of the striosomal system impossible or, at best, problematic.

Nevertheless, a strong and reliable clue from imaging the striatum in HD patients has been derived from positron emission tomography (PET) studies. PET studies have identified reduced striatal phosphodiesterase 10A (PDE10A) as one of the earliest detectable markers in premanifest HD [15,16]. To date, no studies have examined PDE10A in the context of the striosome–matrix compartmentalization, which may represent a novel perspective for understanding its role in HD. This reduction precedes declines in dopamine D1 (Drd1) and Drd2 receptors [15,17], with PDE10A expression decreased by 25–35% approximately 25 years before symptom onset. The reduction is primarily in the dorsal sensorimotor striatum and correlates with disease burden and severity [15,17]. PDE10A, highly expressed in SPNs, regulates cyclic AMP (cAMP) and cyclic GMP (cGMP), with a ~20-fold higher affinity for cAMP [18,19,20]. Both heterozygous and bi-allelic mutations in *PDE10A* cause childhood-onset chorea [21,22,23], suggesting that PDE10A deficiency and consequent cAMP dysregulation might contribute to the pathogenesis of movement disorders [24].

A second lead from the clinic concerns the olfactory type G-protein α subunit (Gα_olf_), encoded by the *GNAL* gene, which has been implicated in hereditary dystonia [25,26,27,28,29,30,31,32,33]. Gα_olf_ is highly concentrated in the striatum and couples positively with Drd1 and adenosine 2A receptors (A2A) in SPNs, thereby increasing intracellular cAMP levels upon receptor stimulation [34,35]. Notably, Gα_olf_ is expressed in both the striosome and matrix compartments, with relatively higher levels in the striosome compartment [36,37]. This compartmentalized expression raises the possibility that alterations in Gα_olf_ signaling might differentially affect striosomal and matrix SPNs, potentially contributing to movement disorders through imbalanced cAMP regulation. Striosome–matrix differences in neuropathology have been linked to hyperkinetic disorders such as X-linked dystonia parkinsonism (XDP) [38], levodopa-induced dyskinesia [37,39], and HD [5,7,8,9,11,12]. Both *PDE10A* and *GNAL* mutations disrupt cAMP signaling; therefore, we reasoned that changes related to cAMP regulation in the striosome and matrix compartments could induce alterations in both the striosomal pathways and the canonical direct–indirect pathways, ultimately leading to movement disorders [14,24,40,41].

Given the lack of human brain imaging to visualize striosomes, we turned to immunohistochemical staining, a traditional method but still one of the best methods to define the striosome compartment. We have recently reported that mu-opioid receptor 1 (MOR1) in the striatum, mainly starting in the striosome compartment, is strikingly and progressively upregulated across ages in the Q175 knock-in (Q175KI) mouse model of HD [42]. Here, we undertook further immunohistochemical staining using Q175KI mice across three ages (3-, 6-, and 12-month-old). We found that Drd1, Drd2, A2A, PDE10A, and Gα_olf_ significantly decreased, especially in the striosomes in the Q175KI mice compared to the wildtype (WT) mice. This mapping leaves MOR1 as the sole upregulated receptor of this broad group. The spatio-temporal alteration in these molecules, although here documented only in a mouse HD model, could yield new insight into the pathogenesis and pathophysiology of HD by demonstrating visible evidence of abnormalities in cAMP-related molecules, and the differential expression of these abnormalities in striosomes.

## 2. Results

### 2.1. Striosomal Segmentation Using Deep Learning by a Fully Convolutional Residual Neural Network U-Net

A performance comparison between the two methods is presented in Supplementary Table S1. Data augmentation using vertical flipping only improved model performance and was compared to training with additional augmentation. The augmentation strategy was, on the basis of this analysis, adopted for subsequent experiments. Representative images are shown in Supplementary Figure S1A–C.

Model stability assessed as the lowest cross-entropy loss was observed at 69 epochs when using vertical flipping without additional augmentation (test accuracy: 0.97965556; test loss: 0.05494046). When vertical flipping was combined with further augmentation, optimal performance was achieved at 44 epochs (test accuracy: 0.97895205; test loss: 0.055600364). These findings suggest that data augmentation with vertical flipping only contributes to the improved training efficiency and generalization performance of the model.

### 2.2. MOR1 Is Upregulated in the Striatum of the Q175KI Mice

**Figure 1:**
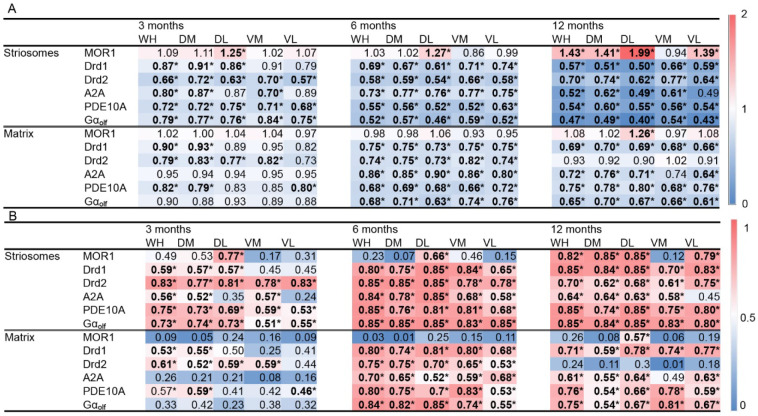
Heatmaps of changes in molecular expression in each striatal segment. The top heatmap (**A**) shows relative intensity, calculated as the ratio of Q175KI to WT values for each segment and molecule. The bottom heatmap (**B**) demonstrates effect size (Cohen’s *r*) for each corresponding segment. Asterisks with values in boldface indicate statistical significance with Kruskal–Wallis tests followed by pairwise Mann–Whitney *U*-tests. Comparisons were specifically made between WT and Q175KI groups (* *p* < 0.005 after Bonferroni corrections). WH: whole, DM: dorsomedial, DL: dorsolateral, VM: ventromedial, and VL: ventrolateral caudoputamen.

**Figure 2:**
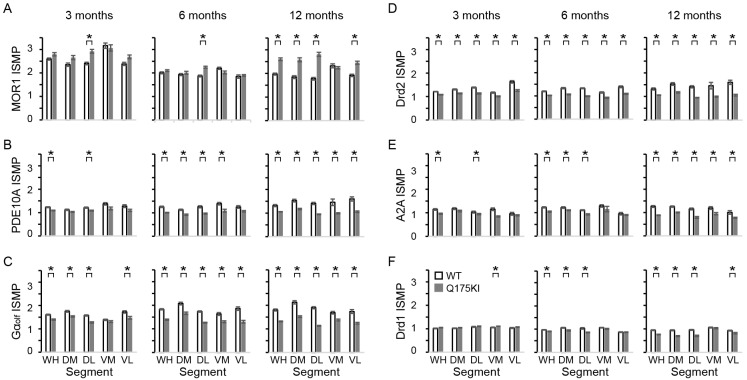
ISMP ratio of MOR1 (**A**), PDE10A (**B**), Gα_olf_ (**C**), Drd2 (**D**), A2A (**E**), and Drd1 (**F**) in WT and Q175 mice across age (3, 6, 12 months). Asterisks indicate statistical significance with Kruskal–Wallis tests followed by pairwise Mann–Whitney *U*-tests (* *p* < 0.005 after Bonferroni corrections).

Confirming our earlier finding [42], MOR1 immunoreactivity was upregulated in the caudoputamen of the Q175KI mice compared to the WT littermate (Figure 1). Figure 1A illustrates the relative ratio (RR) of the mean optical intensity in the Q175KI caudoputamen compared to the WT mice across individual segments and molecules, presenting both as numerical values and a heatmap. Figure 1B displays the corresponding effect sizes derived from these comparisons, providing a quantitative measure of the magnitude of immunoreactivity differences. The upregulation was predominant in the striosome compartment, especially at 12 months old. Compared to the WT mice, the Q175KI mice showed increased intensity of MOR1 labeling in the striosome compartment at 3 and 6 months in the dorsolateral (DL) segment, and at 12 months in the entire ‘whole’ (WH), dorsomedial (DM), dorsolateral (DL), and ventrolateral (VL) segments. In the matrix compartment, the increase was limited to the 12-month DL segment. The index of striosome-to-matrix predominance (ISMP), defined as the ratio of optical intensity in the striosome compartment to that in the matrix compartment (ISMP = [optical intensity in striosomes]/[optical intensity in matrix]), increased in the 3- and 6-month DL, and 12-month WH, DM, DL, and VL segments (Figure 2A). These results indicated that MOR1 expression in the striosomes increased in the DL segment as early as 3 months of age, and at the entire dorsal striatum except the ventromedial (VM) segment at 12 months old. MOR1 expression in the matrix compartment began to increase at 12 months old, as we also observed earlier [42]. This confirmation gives strength to this remarkable upregulation of the MOR1 labeling. As shown in Figure 1, MOR1 was the only molecule of those tested that progressively upregulated its expression in the Q175KI mice.

### 2.3. PDE10A Immunoreactivity over Age

#### 2.3.1. PDE10A Expression Is Relatively Enhanced in the Striosome Compartment in Naïve/WT Mouse and Naïve Monkey

**Figure 3:**
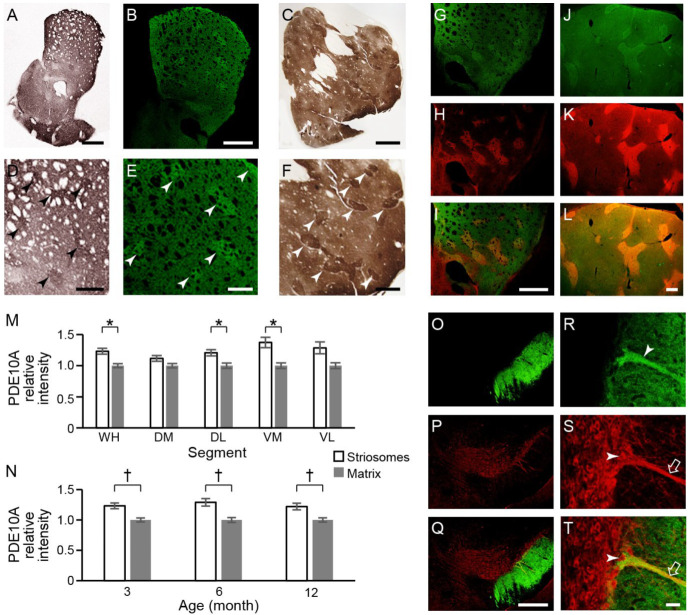
Compartmental distribution of PDE10A labeling in the caudoputamen of the mouse and monkey, and its regional expression in the substantia nigra of the naïve mouse. (**A**–**F**): Microscopic images show DAB staining of mouse (**A**,**D**) and monkey (**C**,**F**) caudoputamen, TSA staining of mouse (**B**,**E**) caudoputamen with low (**A**–**C**) and high (**D**–**F**) magnification. PDE10A showed stronger immunoreactivity in the dorsal caudoputamen and was slightly enriched in the striosome compartment. Arrowheads in (**D**–**F**) indicate striosomes. (**G**–**I**): PDE10A labeling in the mouse caudoputamen (**G**), the same section double-stained with MOR1 (**H**), and merged image (**I**). (**J**–**L**): PDE10A labeling in the monkey caudoputamen (**J**), KChIP1 labeling (**K**), and the merged image (**L**). (**M**,**N**): Densitometric analysis showed that PDE10A immunolabeling in the striosomes is significantly higher than that in the matrix in the WH, DL, and VM segments of a 3-month-old naïve mouse (**M**), and in the WH segment across all ages in WT mice (**N**). Kruskal–Wallis tests followed by pairwise Mann–Whitney *U*-tests (* *p* < 0.005 in (**M**) and † *p* < 0.0167 in (**N**) after Bonferroni corrections). (**O**–**T**): At the level of substantia nigra, PDE10A staining showed massive fiber staining in the SNr (**O**) with some PDE10A-positive fiber passing through substantia nigra reticulata ((**R**); arrowhead) and directly attached to the dendron bouquet positive for tyrosine hydrogenase ((**S**,**T**); arrow) of dopamine-containing neurons ((**S**,**T**); arrowhead). (**P**,**S**) show tyrosine hydrogenase staining in SNc, and (**Q**,**T**) show merged images. Scale bars: 200 µm (**A**–**C**,**I**,**L**,**Q**), 100 µm (**D**–**F**), and 20 µm (**T**).

Immunizing peptide-blocking assay showed that the labeling of PDE10A was blocked when the primary antibody was preabsorbed with 0.05 µg/mL and 0.5 µg/mL PDE10A peptide antigen. At the concentration of 0.5 µg/mL, the PDE10A labeling was completely blocked and rendered equivalent to the level of negative control staining (Supplementary Figure S2). PDE10A was abundant in the caudoputamen of both the mice and macaque monkeys (Figure 3A–F). The striosome compartment’s immunoreactivity appeared to be slightly stronger than that of the surrounding matrix. Double immunostaining of PDE10A and MOR1 in naïve mice demonstrated this slight enrichment of PDE10A expression in the striosome compartment (Figure 3G–I). In the caudoputamen of macaque monkeys, PDE10A also exhibited higher striosomal expression (Figure 3C,F), which was further confirmed by double immunostaining with Kv channel-interacting protein1 (KChIP1)—a reliable marker for striosomes in primates (Figure 3J–L) [43]. These visual impressions were confirmed by the densitometric analyses of 3-month-old naïve mice. The PDE10A labeling was slightly but significantly stronger in the striosomes than in the matrix in the WH, DL, and VM segments (*n* = 16, Figure 3M). The PDE10A labeling in the WH segment of the WT mice was stronger in the striosomes compared to the matrix across 3, 6, and 12 months of age (*n* = 16, Figure 3N). At the level of the substantia nigra, we found massive fiber staining of PDE10A in the substantia nigra pars reticulata (SNr) (Figure 3O) but almost no immunoreactivity in the substantia nigra pars compacta (SNc) (Figure 3O–Q). High magnification fields showed that some PDE10A-positive fibers ran through the SNr (Figure 3R) and eventually combined with the dendron bouquets that extend into the SNr (Figure 3S,T). Striosome–dendron bouquets represent structurally and functionally distinct striatonigral input–output formations consisting of dense arborizations of striosomal fibers that converge onto bundled ventrally extending dendrites of dopamine-containing neurons of the SNc [44,45]. The striosomal fibers tightly interwoven with dopaminergic dendrites can block electrical activity of the dopamine neuron in slice preparations, with following rebound excitation [45]. Consistent with the bouquet architecture, our dual immunostaining for PDE10A and tyrosine hydroxylase (TH) demonstrated direct appositions between striosomal axons and dopaminergic dendrites (Figure 3T). These findings indicate that PDE10A-immunopositive SPNs in the caudoputamen might not only send fibers to the SNr but, as part of this projection, influence the dopaminergic SNc dendron bouquet neurons.

#### 2.3.2. PDE10A Exhibited Decreased Expression in Q175KI Mice

**Figure 4:**
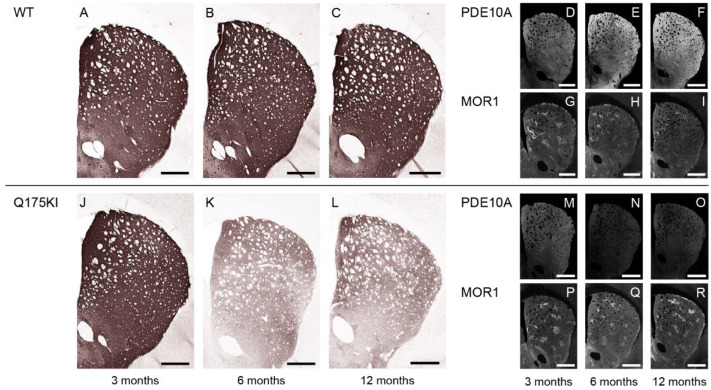
Microscopic images of PDE10A labeling at 3, 6, and 12 months. (**A**–**C**,**J**–**L**): DAB-stained caudoputamen showing PDE10A in WT (**A**–**C**) and Q175KI (**J**–**L**) mice at 3, 6, and 12 months of age. (**D**–**I**,**M**–**R**): TSA-stained caudoputamen showing PDE10A (**D**–**F**,**M**–**O**) double-stained with MOR1 (**G**–**I**,**P**–**R**) in WT and Q175KI mice across age groups. Scale bars: 500 µm.

We examined PDE10A expression in the WT and Q175 mice across 3, 6, and 12 months of age. PDE10A immunoreactivity decreased across all ages in all segments in the caudoputamen of the Q175KI mice except the DL and VM segments in the matrix compartment at 3 months of age (Figure 1 and Figure 4). The ISMP ratios were decreased in all but the DM, VM, and VL segments at 3 months and the VL segment at 6 months (Figure 2B).

### 2.4. Gα_olf_ and Dopamine D2 Receptor Expression Largely Decreased as Early as 3 Months of Age

**Figure 5:**
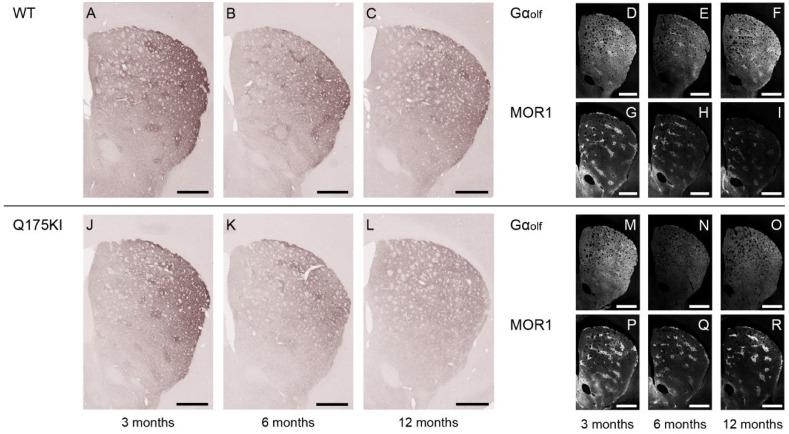
Microscopic images of Gα_olf_ labeling across 3, 6, and 12 months. (**A**–**C**,**J**–**L**): DAB-stained caudoputamen showing Gα_olf_ in WT (**A**–**C**) and Q175KI (**J**–**L**) mice at 3, 6, and 12 months of age. (**D**–**I**,**M**–**R**): TSA-stained caudoputamen showing Gα_olf_ (**D**–**F**,**M**–**O**) double-stained with MOR1 (**G**–**I**,**P**–**R**) in WT and Q175KI mice across age groups. Scale bars: 500 µm.

**Figure 6:**
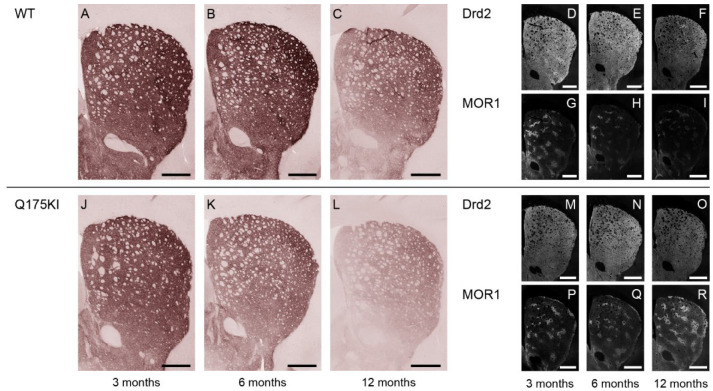
Microscopic images of Drd2 labeling across 3, 6, and 12 months. (**A**–**C**,**J**–**L**): DAB-stained caudoputamen showing Drd2 in WT (**A**–**C**) and Q175KI (**J**–**L**) mice at 3, 6, and 12 months of age. (**D**–**I**,**M**–**R**): TSA-stained caudoputamen showing Drd2 (**D**–**F**,**M**–**O**) double-stained with MOR1 (**G**–**I**,**P**–**R**) in WT and Q175KI mice across age groups. Scale bars: 500 µm.

Gα_olf_ expression decreased across all ages in all segments except in the matrix compartment in the 3-month Q175KI mice (Figure 1 and Figure 5). The ISMP ratios decreased across all ages in all segments in the striatum except for the 3-month VM segment (Figure 2C). Drd2 expression in the striosome compartment decreased in all segments of the caudoputamen across age groups. Regarding the matrix compartment, Drd2 decreased in the WH, DM, DL, and VM segments at 3 months, and in all segments at 6 months but not further at 12 months (Figure 1 and Figure 6). The ISMPs decreased at all ages in all segments (Figure 2D).

### 2.5. Adenosine 2A and Dopamine D1 Receptor Expression Exhibited Relatively Gradual Decreases in Q175KI Mice Through Aging

**Figure 7:**
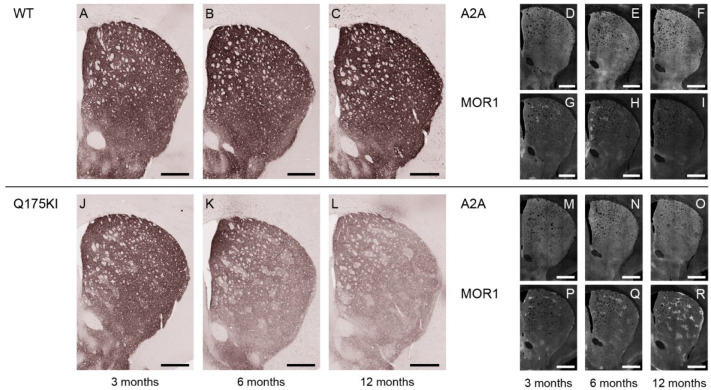
Microscopic images of A2A labeling across 3, 6, and 12 months. (**A**–**C**,**J**–**L**): DAB-stained caudoputamen showing A2A in WT (**A**–**C**) and Q175KI (**J**–**L**) mice at 3, 6, and 12 months of age. (**D**–**I**,**M**–**R**): TSA-stained caudoputamen showing A2A (**D**–**F**,**M**–**O**) double-stained with MOR1 (**G**–**I**,**P**–**R**) in WT and Q175KI mice across age groups. Scale bars: 500 µm.

**Figure 8:**
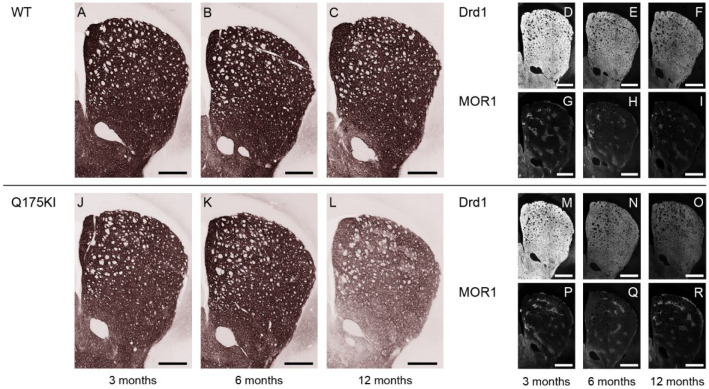
Microscopic images of Drd1 labeling across 3, 6, and 12 months. (**A**–**C**,**J**–**L**): DAB-stained caudoputamen showing Drd1 in WT (**A**–**C**) and Q175KI (**J**–**L**) mice at 3, 6, and 12 months of age. (**D**–**I**,**M**–**R**): TSA-stained caudoputamen showing Drd1 (**D**–**F**,**M**–**O**) double-stained with MOR1 (**G**–**I**,**P**–**R**) in WT and Q175KI mice across age groups. Scale bars: 500 µm.

A2A expression in the striosome compartment was decreased in the WH, DM, and VM segments at 3 months, and in all segments at 6 and 12 months in the Q175KI mice caudoputamen (Figure 1). In the matrix compartment, A2A expression was decreased in all segments at 6 and 12 months in the Q175KI mice caudoputamen. Notably, A2A decreased in the striosomes in the medial (DM and VM) caudoputamen at 3 months of age and then spread to the lateral (DL and VL) caudoputamen at 6 months (Figure 1 and Figure 7). The ISMP ratio decreased in the WH and VM segments at 3 months, WH, DM, and DL at 6 months, and all segments at 12 months in the Q175KI mice (Figure 2E). Drd1 expression decreased across all ages in all segments except the striosome compartment of VM and VL and the matrix compartment of the DL, VM, and VL segments in 3-month-old Q175KI mice (Figure 1 and Figure 8). Compared to the decline in PDE10A, Gα_olf_, and Drd2, the decline in A2A and Drd1 expression at 3 months was relatively limited in both magnitude and effect size (Figure 1). Drd1 ISMP increased in the 3-month VM and decreased in the 6-month WH, DM, and DL and in the 12-month WH, DM, DL, and VL segments (Figure 2F).

## 3. Discussion

In this study, we developed an unbiased machine learning algorithm to measure quantitatively immunohistochemically detectable signals for a panel of major receptors and second messengers across distributed sectors of the striatum of Q175KI mice and their littermate WT mice, distinguishing the striosomes and matrix automatically. Based on established thresholds in image segmentation, the obtained metrics indicate favorable performance. The IoU of 0.6898 approaches the commonly accepted 0.7 threshold, suggesting good spatial overlap. Precision (0.7497) reflects a low false positive rate, while Recall (0.8864) exceeds the desirable 0.8 threshold, indicating high sensitivity. The Dice coefficient (0.8099) falls within the 0.7–0.9 range typically interpreted as high segmentation accuracy. These results align with the standards reported in the prior literature [46,47]. The slightly lower IoU likely reflects boundary-level discrepancies and peripheral over-segmentation, a known issue in anatomically complex regions.

This improved method allowed consistent, comparable measurements to be made across different ages. We concentrated on signaling molecules affecting cAMP processing and documented here decreases in PDE10A, Gα_olf_, A2AR, Drd1, and Drd2 expressions in the Q175KI mice compared to WT, detectable as early as 3 months of age (Figure 1). Their changes lasted long term, up to 12 months of age, which indicates that downregulation of mRNA levels due to the transcriptional dysregulation evoked by mutant huntingtin protein plus the post-translation compensatory reactions induced long-lasting changes in the relative status of these cAMP-related molecules as the mice harboring the huntingtin mutation aged. In the DL segment, the so-called sensorimotor striatum, the striosome compartment was affected in the earliest stage. We suggest as a working hypothesis that the imbalance within the striosome–matrix compartmental system could contribute to the appearance of movement dysfunction [48], and hypothesize that some of the early striosomal changes might also contribute to early signs of mood disorders.

Based on our findings, we consider the following hypothetical scenario as worth further investigation. When compensatory mechanisms fail to sustain cAMP signaling, early impairment may occur in Drd2-SPNs within the striosomes, followed by Drd1-SPNs, potentially altering dopamine dynamics via the striosomal circuit. Concurrently, matrix Drd2-SPN and Drd1-SPN dysfunction may disrupt indirect and direct pathways, and together with dopamine fluctuations, these circuit-level changes could underlie the emergence of parkinsonism and chorea. Striatal cAMP levels were already reduced by approximately 50% in HdhQ111 mice during the symptomatic phase (5–16 months), indicating a progressive decline preceding overt behavioral manifestations [49].

As noted by Deng et al., striatal neuron loss—a hallmark of clinical Huntington’s disease (HD)—is not observed in Q175KI mice up to 18 months of age [50]. Furthermore, profound motor impairments typical of clinical HD are not present in 6–12-month Q175KI mice [50,51]. Smith et al. similarly reported that although synaptic and axonal transport protein abnormalities emerge as early as 6 months, overt striatal atrophy and SPN degeneration become evident only between 12 and 16 months [52]. Based on CAP100 scoring and disease progression models, 3–12-month Q175KI mice are more appropriately considered analogous to the preclinical phase of human HD [53]. Here, we have highlighted attempts to estimate signaling losses that have not led to neuronal death.

### 3.1. Unique Status of MOR1 Protein Expression in HD Mutants

The calculated ratios of striosome to matrix expression (ISMP values) demonstrated that the balance between the striosome and matrix compartments progressively decreased in all molecules except MOR1 (Figure 1). These decreases stood in sharp contrast to the upregulation of MOR1 expression: the MOR1 ISMP ratios increased, especially at 12 months of age (Figure 1). As seen by our results, the increase in MOR1 and the decrease in Drd1 are slow compared to those of the other molecules tested throughout the ages, indicating that alterations in these two molecules also might stem from different mechanisms than the others. The VM segment presented a different set of values, compared to the dorsal striatum, as previously noted [42]. In the VM segment, the Drd1 ISMP increased, and the Drd2 and A2A ISMP ratios decreased at 3 months of age. The Drd1 ISMP did not change at 6 and 12 months of age. MOR1 immunoreactivity and its ISMP ratios did not change throughout the 12-month age limit of the study, despite the fact that the other molecules were decreasing in this segment (Figure 1 and Figure 2). Again, MOR1 was a standout, and furthermore, different mechanisms producing the molecular changes are likely for the dorsal and ventral striatum, a recurring theme in the neuropathology of striatum-based movement disorders. One possibility to account for the MOR1 upregulation observed here is that it might be correlated with excessive dopamine signaling, as occurs in HD. Clinical evidence indicates that dopamine-depleting agents such as tetrabenazine can be effective for treating chorea in HD patients, suggesting abnormally elevated striatal dopamine release in this condition. As MOR1 is normally enriched in striosomal Drd1-type SPNs of the direct pathway, this cell-type-specific and compartment-specific regulation could contribute to the pronounced changes in both Drd1 and MOR1 [54]. Compatible with this possibility, studies in Parkinson’s disease—a condition characterized by striatal dopamine depletion—have shown a reduction in striatal MOR1 expression [55].

If dopamine upregulation were to occur first, a compensatory reaction would be expected to suppress dopamine release by downregulating MOR1 in Drd1-type SPNs [45,54]. Therefore, the observed MOR1 upregulation might precede dopamine elevation, possibly triggered by the dysfunction of Drd2-type SPNs, leading to a reduction in enkephalin—the endogenous ligand for MOR1 [42]. Such a sequence would suggest that MOR1 upregulation may not be a direct response to dopamine excess, but rather, a consequence of impaired opioid signaling due to enkephalin deficiency. If this alternative were the case, then DAMGO—a selective MOR1 agonist—may reduce MOR1 expression and potentially alleviate chorea by suppressing dopamine release.

### 3.2. Clues to the Etiology of Observed Molecular Changes Suggested by Clinical Studies

We have some clues to account for these molecular changes in the previous studies of HD. PDE10A inhibition improves HD symptoms in animal models [56,57,58]. Considering that PDE10A has already decreased, the positive effect of further PDE10A reduction indicates that PDE10A decrease is a compensatory reaction. As for Drd2 decrease, Drd2 antagonists are effective for humans [59,60,61,62,63,64,65] and HD model rats [66], indicating compensatory decreases in Drd2 in HD. In other words, a molecule that changes its expression, probably to lessen cAMP, should exist before the decrease in PDE10A and Drd2. Among the molecules examined, Gα_olf_, A2A, and Drd1 are the molecules whose downregulation could decrease the cAMP levels. A human PET study and the current study indicated that Drd1 decreases its expression relatively in the late stage (Figure 1) [15,17]. In addition, Drd1 antagonists rescue symptoms, suggesting that the Drd1 decrease might also be a compensatory reaction [67,68]. In the current study, early A2A decreases are confined to the medial caudoputamen, i.e., the DM and VM segments. Although pharmacological studies targeting A2A in genetically engineered HD mouse models are scarce and the supporting evidence remains limited, certain findings suggest that A2A antagonism may ameliorate emotional and cognitive deficits, potentially linked to the medial caudoputamen [69,70]. Moreover, assuming that Drd1 or A2A expression is initially reduced, previous studies have reported an upregulation of Gα_olf_ under such conditions [71]. The reduction in Drd1 and A2A receptor levels may occur post-translationally as a mechanism to suppress the usage-dependent depletion of Gα_olf_, and may therefore represent a compensatory change.

### 3.3. Hypothesis: Gα_olf_ as a Primary Etiologic Signal of Abnormality in HD Model Mice

According to these working suggestions, Gα_olf_ might be a candidate for the primary change. Gα_olf_ couples with Drd1 and A2A and normally changes its expression depending on its usage [71]. However, in the current case, a decrease in the expression of Gα_olf_ was already observed in 3-month-old Q175KI mice, before the apparent upregulation of MOR1. As we previously hypothesized, dopamine levels parallel the upregulation in MOR1, indicating that the early Gα_olf_ decrease might not be usage-dependent due to upregulation in dopamine levels [42]. An increase in dopamine levels during the early clinical hyperkinetic stage of HD has been reported [72,73,74]. This timeline implies that the early Gα_olf_ decrease might be attributed to transcriptional abnormalities caused by mHtt. If translational Gα_olf_ decreases occur first, they could also induce hypersensitivity of Drd1 and A2A [71]. These hypersensitivities could promote subsequent usage-dependent consumption of Gα_olf_.

In the material analyzed here, the early striosomal changes in all segments were common in the molecules related to Drd2, as identified previously by snRNA-seq analysis [13]. Early loss of Drd2-type SPNs has also been found in HD postmortem studies [3,6,75,76]. The decrease in A2A/Gα_olf_ signaling in the striosome compartment could lead to a subsequent downregulation in Drd2/Gi signaling to compensate for the cAMP decrease. Loss of function in striosomal Drd2-type SPNs could in turn downregulate dopamine release and lead to parkinsonism [45]. To support this notion, parkinsonism is detectable early on in premanifest gene carriers up to two decades before the clinical manifestation of HD symptoms [77]. Based on these considerations, at the very early preclinical stage, administration of a Drd2 antagonist may represent a therapeutic strategy by correcting abnormalities in the striosomal circuitry and indirect pathway associated with Drd2 dysfunction. In addition, a decrease in the Drd1/Gα_olf_ signal in the striosome compartment in later stages could produce upregulation of dopamine release in the striatum via the striosomal pathways [45], and if so, this might induce chorea. Drd1-type SPNs in the striosome compartment are thought to have suppressive reciprocal innervation of the dopamine-containing neurons of the SNc [45]. As for the striosome–dendron bouquets, the complex timing of signals occurs within the substantia nigra itself, and this is a major factor that needs study [45]. We acknowledge that the causal relationships among these changes remain speculative. We should interpret these findings with caution and recognize the need for further experimental validation.

### 3.4. The Potential Role of PDE10A and Changes in cAMP

In regard to clinical work, we realize that research findings on mouse models of HD are imperfect indicators of the status of the HD striatum. They could even be misleading. There is, however, reason to consider our findings in relation not only to HD but to other movement disorders. We found PDE10A to be slightly but significantly more strongly expressed in the striosomes than in the matrix in neurotypical rodents and non-human primates, and found that PDE10A strikingly decreased its expression in the striosome compartment in the Q175KI mice. Childhood-onset familial chorea cases with *PDE10A* mutations and PDE10A reduction in HD are the focus of recent studies in the field of movement disorders [21,22,23,78]. As we have noted, PDE10A is a critical regulator of cAMP in both direct and indirect pathway neurons [19]. Gα_olf_ is a G-protein that couples with dopamine D1 receptors in SPNs and also with adenosine A2A receptors that co-localize with D2 receptors in SPNs [33,34,71]. A mutation of *GNAL*, which encodes Gα_olf_, also induces hereditary dystonia, another form of hyperkinetic movement disorder [25,26,27,28,29,30,31,32,33]. Further, Gα_olf_ is also distributed preferentially in striosomes, and its alteration in striosomes is correlated with levodopa-induced dyskinesia in Parkinson’s disease mouse models [24,37]. These coincidences suggested that dysregulation of the cAMP system in striosomal SPNs could be a key factor in the genesis of hyperkinetic movement disorders including chorea, dystonia, and levodopa-induced dyskinesia [24,79]. Therefore, in cases where disease onset can be genetically predicted, it may be preferable to administer PDE10A inhibitors and/or Drd2 antagonists early in the preclinical phase, which may contribute to delaying the progression of the vicious cycle of neuronal cell death. Although a clinical trial of PDE10A inhibitors in symptomatic HD patients has reported a lack of efficacy [80], the findings of the present study suggest that the timing of intervention may have been too late, occurring after substantial neurodegenerative changes had already taken place.

## 4. Material and Methods

### 4.1. Animals

All experimental protocols involving work with mice were approved by the Committee on Animal Care at the Massachusetts Institute of Technology. For the primary analysis, twenty-four mice, four male and four female heterozygous Q175KI mice, and matching numbers for WT littermates for each of three ages (3, 6, and 12 months postnatal) were used. Additionally, we used six mice each at 2, 6, and 19 months of age. All mice were obtained from Jackson Laboratory (Bar Harbor, ME, USA) or our laboratory colony at MIT.

### 4.2. Immunizing Peptide-Blocking Assay

The immunizing peptide-blocking assay for rabbit polyclonal PDE10A (Creative Diagnostics, Shirley, NY, USA) was performed as described previously [42].

### 4.3. Immunohistochemistry

Immunohistochemistry was performed as described previously [36,37,42,81]. In brief, 20 μm thick sections were cut on a sliding microtome, and free-floating sections were immunohistochemically stained. After a step for blocking endogenous peroxidase activity, sections were incubated in phosphate-buffered saline (PBS) containing 3% bovine serum albumin (BSA) for 60 min and then were incubated in the chosen antibodies for 15 h (Supplementary Table S2). Bound antibody was detected by use of anti-rabbit (Invitrogen, Carlsberg, CA, USA), anti-goat (Vector, Burlingame, CA, USA), or anti-rat (Nichirei, Tokyo, Japan) IgG polymer secondary antibodies, and bound peroxidase was detected by 3,3′-diamonobenzidine (DAB) (Vector, Burlingame, CA, USA) or the TSA system (fluorescein or Cy5) (Perkin Elmer, Shelton, CT, USA). For dual antigen detection of fluorescent reagents, sections were incubated in 0.1 M glycine-HCl (pH 2.2) for 30 min after the first antigen procedures to remove the bound antibody. After several rinses in PBS, sections were incubated for 15 h in PBS containing 3% BSA and rabbit anti-MOR1. Bound antibody was detected the same as the first antibody. The sections from WT and Q175KI mice of the same ages for each target molecule were stained simultaneously.

### 4.4. Immunohistochemical Detection of PDE10A in Monkeys’ Brain

Striatal sections were obtained from two *Macaca mulatta* monkeys housed and treated in accordance with the NIH Guide for the Care and Use of Laboratory Animals, with approval from the MIT Committee on Animal Care. Under deep barbiturate anesthesia (50 mg/kg, i.p.), monkeys were perfused transcardially with 0.9% saline followed by 4% paraformaldehyde in 0.1 M phosphate-buffered 5% sucrose, and post-fixed in phosphate-buffered sucrose. Brains were cryoprotected in 20% glycerol for 3 days and coronally sectioned at 40 µm using a freezing microtome. Immunostaining was performed using the free-floating method. For PDE10A detection, the PBTA protocol was applied as follows: sections were incubated with anti-PDE10A antibody (1:100,000) for 2 days, followed by anti-rabbit polymer reagent (Invitrogen) and TSA amplification (Perkin Elmer) [81]. Signal was visualized with nickel-enhanced DAB and 0.01% H_2_O_2_. For dual antigen detection, sections were processed similarly to mouse tissue, except using mouse anti-KChIP1 antibody (1:2000; NeuroMab, Davis, CA, USA) and anti-mouse polymer secondary antibody.

### 4.5. Striosomal Segmentation

To perform the segmentation of striatal compartments automatically and objectively, we developed an algorithm using deep learning by a fully convolutional residual neural network U-Net [82]. We randomly selected 176 MOR1 fluorescent images from 2-, 3-, 6-, 9-, 12-, and 19-month-old WT and Q175KI mice as training images (12,000 × 9000 pixels). Firstly, two authors (RM and JF) manually defined all of the striosomes visible, and then fibers and vessels, with the assistance of Otsu’s method that performed automatic image thresholding [83]. The background intensities were set on the slide grass outside the tissue. Then, the dorsal striatum was divided into 2376 training images (512 × 512 pixels for each image). We tested two methods, augmentation with vertical flip only, or with vertical flip combined with additional augmentation techniques (bright-ness, hue, and saturation) for each image. The evaluation was performed using 8 randomly selected images (total 24 images, 48 striatum) from 3-, 6-, and 12-month-old WT and Q175KI mice. The batch size of the training set was 32, and the total epoch was 160. The training rate was set as 0.85. Manual segmentation of striosomes was conducted and quality-checked by an experienced investigator (RM), providing the gold standard reference for evaluating the algorithm. Intersection over Union (IoU), Precision, Recall (Sensitivity), and Dice coefficient were calculated on the test set. IoU and Dice quantify the spatial overlap between predicted and reference regions, while Precision and Recall provide information on false positives and false negatives, respectively.

### 4.6. Digital Images and Densitometry

Microscopic images were captured with a TissueFAXS whole-slide scanning system equipped with a Zeiss Axio Imager Z2 fluorescence microscope (Zeiss, Oberkochen, Germany) and TissueFAXS image acquisition software (version 7) as described before [42]. The acquired images were processed and analyzed with MATLAB software version R2020b (The MathWorks, Inc., Natick, MA, USA). The optical density of each molecule’s immunolabeling was entered as gray levels on non-colored digital images. Each molecule’s labeling was measured in the striosomal and matrix subfields of the striatum demarcated by rabbit monoclonal anti-MOR1 antibody for each mouse (n = 8, each with both hemispheres, yielding 16 caudoputamen samples) across each age (3, 6, and 12 months). The levels of the caudoputamen, coronal sections from +0.98 to +1.34 mm rostral to the bregma [84] were selected for each WT and Q175KI mouse. We arranged 24 serial sections (20 µm thick) in a 96-well plate for each mouse and selected the brain region of these coordinates as nearly identical as possible for immunohistochemical staining of each molecule. Four segments in the caudoputamen (DM: dorsomedial, DL: dorsolateral, VM: ventromedial, and VL: ventrolateral) were manually defined [42]. We defined “WH” as the whole area of the caudoputamen, which was the sum of these four segments. Then, the MOR1-immunopositive regions were objectively defined by deep learning using the U-net architecture. The MOR1-immunopositive striosomal regions were superimposed on the double-stained immunohistochemical images of each molecule. The densitometry analyses were performed for the striosome and matrix compartments, excluding fiber bundles. Segments exhibiting poor staining or tissue loss were excluded from subsequent analyses following visual inspection of the histological images. Each segment’s relative intensity (RI) was calculated as a ratio of the mean optical intensity in Q175KI mice relative to the mean for WT for each segment and age:

RI = (mean optical intensity of Q175KI mice)/(mean optical intensity of WT mice)(1)

The relative ratio of striosomal and matrix intensities, measured as an index of striosome-to-matrix predominance (ISMP), was calculated in each segment of the same animals [42,48]:

ISMP = (optical intensity in striosomes)/(optical intensity in matrix)(2)

### 4.7. Statistical Analysis

All quantitative data were expressed as means ± SEM values. The Kruskal–Wallis tests followed by pairwise Mann–Whitney *U*-tests with Bonferroni corrections were used. *p*-values less than 0.05 were considered statistically significant. The effect size was expressed using Cohen’s *r*, with conventional thresholds: *r* = 0.1 for small, *r* = 0.3 for medium, and *r* = 0.5 for large effects. For sample size estimation, we used G*Power 3.1 with the assumptions of a medium effect size (Cohen’s *d* = 0.5), an alpha error of 0.05, and a statistical power of 0.8. Based on these parameters, 43 samples were estimated to be required for each group (WT and Q175KI mice). Assuming that three striosomes can be detected in each subregion of the caudoputamen, we calculated that six striosomes could be obtained bilaterally per mouse, resulting in a total of 48 samples from eight mice.

## 5. Conclusions

Here, we report a coordinated study demonstrating massive and chronic changes in the expression of multiple molecules in the Q175KI mouse striatum. All but MOR1 progressively decreased their expression, primarily in the striosomes, and diminished their trans-compartmental differential expressions. This preclinical imbalance of striosome–matrix cAMP-related molecules might be a key to delineating HD symptoms. This model could also help to examine mechanisms that could underlie the greater vulnerability of striosomal SPNs relative to those in the matrix.

A significant limitation of our study is the small number of molecules analyzed due to the limited number of sections per mouse, which might not be enough to clarify the precise pathological changes. Although our conclusions are based on a single mouse model, we acknowledge that further validation using additional models and human tissue will be essential to confirm the generalizability and translational relevance of these findings. This study focuses on molecular alterations, and does not include behavioral, physiological, or in vivo imaging data to directly link these changes to functional outcomes. Future investigations incorporating such approaches will be critical to establish causal relationships and enhance translational relevance. As noted in the snRNA-seq study of Q175KI mice, there is multiplexing of transcriptional changes in direct-pathway (Drd1-type) and indirect-pathway (Drd2-type) SPNs [13]. Abnormalities in the striosome compartment have further been linked to some neuropsychiatric disorders [14]. Currently, several PDE10A inhibitors are being examined as therapeutics in patients with schizophrenia [85,86,87,88]. These trials might shed light on the interaction between the cAMP system in the striosome compartment and psychiatric disorders [89,90]. Our findings join these studies in suggesting that targeting altered cAMP levels in the striosome compartment during the early preclinical stage of the disease could improve symptoms in patients with HD.

## Data Availability

The original contributions presented in this study are included in the article/Supplementary Materials. The original images and raw data can be accessed via DOI:10.5281/zenodo.15878997. Further inquiries can be directed to the corresponding author.

## Supplementary Materials

The following supporting information can be downloaded at: https://www.mdpi.com/article/10.3390/ijms26178573/s1.

## References

[1] Ferrante R.J., Kowall N.W., Beal M.F., Richardson E.P., Bird E.D., Martin J.B. (1985). Selective Sparing of a Class of Striatal Neurons in Huntington’s Disease. Science.

[2] Ferrante R.J., Flint Beal M., Kowall N.W., Richardson E.P., Martin J.B. (1987). Sparing of Acetylcholinesterase-Containing Striatal Neurons in Huntington’s Disease. Brain Res..

[3] Reiner A., Albin R.L., Anderson K.D., D’Amato C.J., Penney J.B., Young A.B. (1988). Differential Loss of Striatal Projection Neurons in Huntington Disease. Proc. Natl. Acad. Sci. USA.

[4] Reiner A., Shelby E., Wang H., DeMarch Z., Deng Y., Guley N.H., Hogg V., Roxburgh R., Tippett L.J., Waldvogel H.J. (2013). Striatal Parvalbuminergic Neurons Are Lost in Huntington’s Disease: Implications for Dystonia. Mov. Disord..

[5] Goto S., Hirano A., Rojas-Corona R.R. (1989). An Immunohistochemical Investigation of the Human Neostriatum in Huntington’s Disease. Ann. Neurol..

[6] Albin R.L., Reiner A., Anderson K.D., Penney J.B., Young A.B. (1990). Striatal and Nigral Neuron Subpopulations in Rigid Huntington’s Disease: Implications for the Functional Anatomy of Chorea and Rigidity-Akinesia. Ann. Neurol..

[7] Morton A.J., Nicholson L.F.B., Faull R.L.M. (1993). Compartmental Loss of NADPH Diaphorase in the Neuropil of the Human Striatum in Huntington’s Disease. Neuroscience.

[8] Hedreen J.C., Folstein S.E. (1995). Early Loss of Neostriatal Striosome Neurons in Huntington’s Disease. J. Neuropathol. Exp. Neurol..

[9] Augood S.J., Faull R.L.M., Love D.R., Emson P.C. (1996). Reduction in Enkephalin and Substance P Messenger RNA in the Striatum of Early Grade Huntington’s Disease: A Detailed Cellularin Situ Hybridization Study. Neuroscience.

[10] Cicchetti F., Prensa L., Wu Y., Parent A. (2000). Chemical Anatomy of Striatal Interneurons in Normal Individuals and in Patients with Huntington’s Disease. Brain Res. Rev..

[11] Tippett L.J., Waldvogel H.J., Thomas S.J., Hogg V.M., Roon-Mom W.V., Synek B.J., Graybiel A.M., Faull R.L.M. (2007). Striosomes and Mood Dysfunction in Huntington’s Disease. Brain.

[12] Hedreen J.C., Berretta S., White Iii C.L. (2024). Postmortem Neuropathology in Early Huntington Disease. J. Neuropathol. Exp. Neurol..

[13] Matsushima A., Pineda S.S., Crittenden J.R., Lee H., Galani K., Mantero J., Tombaugh G., Kellis M., Heiman M., Graybiel A.M. (2023). Transcriptional Vulnerabilities of Striatal Neurons in Human and Rodent Models of Huntington’s Disease. Nat. Commun..

[14] Crittenden J.R., Graybiel A.M. (2011). Basal Ganglia Disorders Associated with Imbalances in the Striatal Striosome and Matrix Compartments. Front. Neuroanat..

[15] Niccolini F., Haider S., Reis Marques T., Muhlert N., Tziortzi A.C., Searle G.E., Natesan S., Piccini P., Kapur S., Rabiner E.A. (2015). Altered PDE10A Expression Detectable Early before Symptomatic Onset in Huntington’s Disease. Brain.

[16] Wilson H., Niccolini F., Haider S., Marques T.R., Pagano G., Coello C., Natesan S., Kapur S., Rabiner E.A., Gunn R.N. (2016). Loss of Extra-Striatal Phosphodiesterase 10A Expression in Early Premanifest Huntington’s Disease Gene Carriers. J. Neurol. Sci..

[17] Russell D.S., Barret O., Jennings D.L., Friedman J.H., Tamagnan G.D., Thomae D., Alagille D., Morley T.J., Papin C., Papapetropoulos S. (2014). The Phosphodiesterase 10 Positron Emission Tomography Tracer, [^18^ F]MNI-659, as a Novel Biomarker for Early Huntington Disease. JAMA Neurol..

[18] Kotera J., Fujishige K., Yuasa K., Omori K. (1999). Characterization and Phosphorylation of PDE10A2, a Novel Alternative Splice Variant of Human Phosphodiesterase That Hydrolyzes cAMP and cGMP. Biochem. Biophys. Res. Commun..

[19] Nishi A., Kuroiwa M., Miller D.B., O’Callaghan J.P., Bateup H.S., Shuto T., Sotogaku N., Fukuda T., Heintz N., Greengard P. (2008). Distinct Roles of PDE4 and PDE10A in the Regulation of cAMP/PKA Signaling in the Striatum. J. Neurosci..

[20] Xie Z., Adamowicz W.O., Eldred W.D., Jakowski A.B., Kleiman R.J., Morton D.G., Stephenson D.T., Strick C.A., Williams R.D., Menniti F.S. (2006). Cellular and Subcellular Localization of PDE10A, a Striatum-Enriched Phosphodiesterase. Neuroscience.

[21] Esposito S., Carecchio M., Tonduti D., Saletti V., Panteghini C., Chiapparini L., Zorzi G., Pantaleoni C., Garavaglia B., Krainc D. (2017). A *PDE10A* de Novo Mutation Causes Childhood-Onset Chorea with Diurnal Fluctuations: *PDE10A* Mutation. Mov. Disord..

[22] Narayanan D.L., Deshpande D., Das Bhowmik A., Varma D.R., Dalal A. (2018). Familial Choreoathetosis Due to Novel Heterozygous Mutation in *PDE10A*. Am. J. Med. Genet..

[23] Miyatake S., Koshimizu E., Shirai I., Kumada S., Nakata Y., Kamemaru A., Nakashima M., Mizuguchi T., Miyake N., Saitsu H. (2018). A Familial Case of *PDE10A*-associated Childhood-onset Chorea with Bilateral Striatal Lesions. Mov. Disord..

[24] Goto S. (2017). Striatal Gαolf/cAMP Signal-Dependent Mechanism to Generate Levodopa-Induced Dyskinesia in Parkinson’s Disease. Front. Cell. Neurosci..

[25] Carecchio M., Panteghini C., Reale C., Barzaghi C., Monti V., Romito L., Sasanelli F., Garavaglia B. (2016). Novel GNAL Mutation with Intra-Familial Clinical Heterogeneity: Expanding the Phenotype. Park. Relat. Disord..

[26] Dobričić V., Kresojević N., Westenberger A., Svetel M., Tomić A., Ralić V., Petrović I., Lukić M.J., Lohmann K., Novaković I. (2014). De Novo Mutation in the *GNAL* Gene Causing Seemingly Sporadic Dystonia in a Serbian Patient: DE NOVO *GNAL* (DYT25) MUTATION. Mov. Disord..

[27] Fuchs T., Saunders-Pullman R., Masuho I., Luciano M.S., Raymond D., Factor S., Lang A.E., Liang T.-W., Trosch R.M., White S. (2013). Mutations in GNAL Cause Primary Torsion Dystonia. Nat. Genet..

[28] Kaur A. (2013). Rare Autosomal Dominant Mutations in *GNAL* Are Associated with Primary Torsion Dystonia: HotSpots. Clin. Genet..

[29] Kumar K.R., Lohmann K., Masuho I., Miyamoto R., Ferbert A., Lohnau T., Kasten M., Hagenah J., Brüggemann N., Graf J. (2014). Mutations in *GNAL*: A Novel Cause of Craniocervical Dystonia. JAMA Neurol..

[30] Masuho I., Fang M., Geng C., Zhang J., Jiang H., Özgul R.K., Yılmaz D.Y., Yalnızoğlu D., Yüksel D., Yarrow A. (2016). Homozygous *GNAL* Mutation Associated with Familial Childhood-Onset Generalized Dystonia. Neurol. Genet..

[31] Miao J., Wan X.-H., Sun Y., Feng J.-C., Cheng F.-B. (2013). Mutation Screening of GNAL Gene in Patients with Primary Dystonia from Northeast China. Park. Relat. Disord..

[32] Putzel G.G., Fuchs T., Battistella G., Rubien-Thomas E., Frucht S.J., Blitzer A., Ozelius L.J., Simonyan K. (2016). *GNAL* Mutation in Isolated Laryngeal Dystonia: *Gnal* in Laryngeal Dystonia. Mov. Disord..

[33] Vemula S.R., Puschmann A., Xiao J., Zhao Y., Rudzińska M., Frei K.P., Truong D.D., Wszolek Z.K., LeDoux M.S. (2013). Role of Gα(Olf) in Familial and Sporadic Adult-Onset Primary Dystonia. Hum. Human. Mol. Genet..

[34] Hervé D. (2011). Identification of a Specific Assembly of the G Protein Golf as a Critical and Regulated Module of Dopamine and Adenosine-Activated cAMP Pathways in the Striatum. Front. Neuroanat..

[35] Corvol J.C., Studler J.M., Schonn J.S., Girault J.A., Hervé D. (2001). Gαolf Is Necessary for Coupling D1 and A2a Receptors to Adenylyl Cyclase in the Striatum: Gαolf-Coupled Striatal Adenylyl Cyclase. J. Neurochem..

[36] Sako W., Morigaki R., Nagahiro S., Kaji R., Goto S. (2010). Olfactory Type G-Protein α Subunit in Striosome-Matrix Dopamine Systems in Adult Mice. Neuroscience.

[37] Morigaki R., Okita S., Goto S. (2017). Dopamine-Induced Changes in Gαolf Protein Levels in Striatonigral and Striatopallidal Medium Spiny Neurons Underlie the Genesis of l-DOPA-Induced Dyskinesia in Parkinsonian Mice. Front. Cell. Neurosci..

[38] Goto S., Lee L.V., Munoz E.L., Tooyama I., Tamiya G., Makino S., Ando S., Dantes M.B., Yamada K., Matsumoto S. (2005). Functional Anatomy of the Basal Ganglia in X-Linked Recessive Dystonia-Parkinsonism. Ann. Neurol..

[39] Crittenden J.R., Cantuti-Castelvetri I., Saka E., Keller-McGandy C.E., Hernandez L.F., Kett L.R., Young A.B., Standaert D.G., Graybiel A.M. (2009). Dysregulation of CalDAG-GEFI and CalDAG-GEFII Predicts the Severity of Motor Side-Effects Induced by Anti-Parkinsonian Therapy. Proc. Natl. Acad. Sci. USA.

[40] Morigaki R., Goto S. (2017). Striatal Vulnerability in Huntington’s Disease: Neuroprotection Versus Neurotoxicity. Brain Sci..

[41] Graybiel A.M., Canales J.J., Capper-Loup C. (2000). Levodopa-Induced Dyskinesias and Dopamine-Dependent Stereotypies: A New Hypothesis. Trends Neurosci..

[42] Morigaki R., Lee J.H., Yoshida T., Wüthrich C., Hu D., Crittenden J.R., Friedman A., Kubota Y., Graybiel A.M. (2020). Spatiotemporal Up-Regulation of Mu Opioid Receptor 1 in Striatum of Mouse Model of Huntington’s Disease Differentially Affecting Caudal and Striosomal Regions. Front. Neuroanat..

[43] Mikula S., Parrish S.K., Trimmer J.S., Jones E.G. (2009). Complete 3-D visualization of primate striosomes by KChIP1 immunostaining. J. Comp. Neurol..

[44] Crittenden J.R., Tillberg P.W., Riad M.H., Shima Y., Gerfen C.R., Curry J., Housman D.E., Nelson S.B., Boyden E.S., Graybiel A.M. (2016). Striosome–Dendron Bouquets Highlight a Unique Striatonigral Circuit Targeting Dopamine-Containing Neurons. Proc. Natl. Acad. Sci. USA.

[45] Lazaridis I., Crittenden J.R., Ahn G., Hirokane K., Wickersham I.R., Yoshida T., Mahar A., Skara V., Loftus J.H., Parvataneni K. (2024). Striosomes Control Dopamine via Dual Pathways Paralleling Canonical Basal Ganglia Circuits. Curr. Biol..

[46] Taha A.A., Hanbury A. (2015). Metrics for evaluating 3D medical image segmentation: Analysis, selection, and tool. BMC Med. Imaging.

[47] Reinke A., Tizabi M.D., Baumgartner M., Eisenmann M., Heckmann-Nötzel D., Kvur A.E., Rädsch T., Sudre C.H., Acion L., Antonelli M. (2024). Understanding metric-related pitfalls in image analysis validation. Nat. Methods.

[48] Canales J.J., Graybiel A.M. (2000). A Measure of Striatal Function Predicts Motor Stereotypy. Nat. Neurosci..

[49] Gines S., Seong I.S., Fossale E., Ivanova E., Trettel F., Gusella J.F., Wheeler V.C., Persichetti F., MacDonald M.E. (2003). Specific progressive cAMP reduction implicates energy deficit in presymptomatic Huntington’s disease knock-in mice. Hum. Mol. Genet..

[50] Deng Y., Wang H., Joji M., Sekhri R., Reiner A. (2021). Progression of basal ganglia pathology in heterozygous Q175 knock-in Huntington’s disease mice. J. Comp. Neurol..

[51] Menalled L.B., Kudwa A.E., Miller S., Fitzpatrick J., Watson-Johnson J., Keating N., Ruiz M., Mushlin R., Alosio W., McConnell K. (2012). Comprehensive behavioral and molecular characterization of a new knock-in mouse model of Huntington’s disease: zQ175. PLoS ONE.

[52] Smith G.A., Rocha E.M., McLean J.R., Hayes M.A., Izen S.C., Isacson O., Hallett P.J. (2014). Progressive axonal transport and synaptic protein changes correlate with behavioral and neuropathological abnormalities in the heterozygous Q175 KI mouse model of Huntington’s disease. Hum. Mol. Genet..

[53] Warner J.H., Long J.D., Mills J.A., Langbehn D.R., Ware J., Mohan A., Sampaio C. (2022). Standardizing the CAP score in Huntington’s disease by predicting age-at-onset. J. Huntingt. Dis..

[54] Cui Y., Ostlund S.B., James A.S., Park C.S., Ge W., Roberts K.W., Mittal N., Murphy N.P., Cepeda C., Kieffer B.L. (2014). Targeted expression of μ-opioid receptors in a subset of striatal direct-pathway neurons restores opiate reward. Nat. Neurosci..

[55] Koizumi H., Morigaki R., Okita S., Nagahiro S., Kaji R., Nakagawa M., Goto S. (2013). Response of striosomal opioid signaling to dopamine depletion in 6-hydroxydopamine-lesioned rat model of Parkinson’s disease: A potential compensatory role. Front. Cell. Neurosci..

[56] Giampà C., Laurenti D., Anzilotti S., Bernardi G., Menniti F.S., Fusco F.R. (2010). Inhibition of the Striatal Specific Phosphodiesterase PDE10A Ameliorates Striatal and Cortical Pathology in R6/2 Mouse Model of Huntington’s Disease. PLoS ONE.

[57] Giralt A., Saavedra A., Carretón O., Arumí H., Tyebji S., Alberch J., Pérez-Navarro E. (2013). PDE10 Inhibition Increases GluA1 and CREB Phosphorylation and Improves Spatial and Recognition Memories in a Huntington’s Disease Mouse Model: Pde10 Inhibition Improves Cognition in Huntington’s Disease. Hippocampus.

[58] Beaumont V., Zhong S., Lin H., Xu W., Bradaia A., Steidl E., Gleyzes M., Wadel K., Buisson B., Padovan-Neto F.E. (2016). Phosphodiesterase 10A Inhibition Improves Cortico-Basal Ganglia Function in Huntington’s Disease Models. Neuron.

[59] Roos R.A.C., Buruma O.J.S., Bruyn G.W., Kemp B., Velde E.A. (1982). Tiapride in the Treatment of Huntington’s Chorea. Acta Neurol. Scand..

[60] Deroover J., Baro F., Bourguignon R.P., Smets P.h. (1984). Tiapride versus Placebo: A Double-Blind Comparative Study in the Management of Huntington’s Chorea. Curr. Med. Res. Opin..

[61] Quinn N., Marsden C.D. (1984). A Double Blind Trial of Sulpiride in Huntington’s Disease and Tardive Dyskinesia. J. Neurol. Neurosurg. Psychiatry.

[62] Koller W.C., Trimble J. (1985). The Gait Abnormality of Huntington’s Disease. Neurology.

[63] Bonuccelli U., Ceravolo R., Maremmani C., Nuti A., Rossi G., Muratorio A. (1994). Clozapine in Huntington’s Chorea. Neurology.

[64] Duff K., Beglinger L.J., O’Rourke M.E., Nopoulos P., Paulson H.L., Paulsen J.S. (2008). Risperidone and the Treatment of Psychiatric, Motor, and Cognitive Symptoms in Huntington’s Disease. Ann. Clin. Psychiatry.

[65] Schultz J.L., Kamholz J.A., Nopoulos P.C., Killoran A. (2019). Comparing Risperidone and Olanzapine to Tetrabenazine for the Management of Chorea in Huntington Disease: An Analysis from the Enroll-HD Database. Mov. Disord. Clin. Pract..

[66] Charvin D., Roze E., Perrin V., Deyts C., Betuing S., Pagès C., Régulier E., Luthi-Carter R., Brouillet E., Déglon N. (2008). Haloperidol Protects Striatal Neurons from Dysfunction Induced by Mutated Huntingtin in Vivo. Neurobiol. Dis..

[67] Tang T.-S., Chen X., Liu J., Bezprozvanny I. (2007). Dopaminergic Signaling and Striatal Neurodegeneration in Huntington’s Disease. J. Neurosci..

[68] André V.M., Cepeda C., Fisher Y.E., Huynh M., Bardakjian N., Singh S., Yang X.W., Levine M.S. (2011). Differential Electrophysiological Changes in Striatal Output Neurons in Huntington’s Disease. J. Neurosci..

[69] Domenici M.R., Scattoni M.L., Martire A., Lastoria G., Potenza R.L., Borioni A., Venerosi A., Calamandrei G., Popoli P. (2007). Behavioral and Electrophysiological Effects of the Adenosine A2A Receptor Antagonist SCH 58261 in R6/2 Huntington’s Disease Mice. Neurobiol. Dis..

[70] Li W., Silva H.B., Real J., Wang Y.M., Rial D., Li P., Payen M.P., Zhou Y., Muller C.E., Tomé A.R. (2015). Inactivation of adenosine A2A receptors reverses working memory deficits at early stages of Huntington’s disease models. Neurobiol. Dis..

[71] Hervé D., Le Moine C., Corvol J.-C., Belluscio L., Ledent C., Fienberg A.A., Jaber M., Studler J.-M., Girault J.-A. (2001). Gα_olf_ Levels Are Regulated by Receptor Usage and Control Dopamine and Adenosine Action in the Striatum. J. Neurosci..

[72] Chen J.Y., Wang E.A., Cepeda C., Levine M.S. (2013). Dopamine imbalance in Huntingoton’s disease: A mechanism for the lack of behavioral flexibility. Front. Neurosci..

[73] Cepeda C., Murphy K.P.S., Parent M., Levine M.S. (2014). The role of dopamine in Huntington’s disease. Prog. Brain Res..

[74] Reiner A., Deng Y.P. (2018). Disrupted striatal neuron inputs and outputs in Huntington’s disease. CNS Neurosci. Ther..

[75] Richfield E.K., Maguire-Zeiss K.A., Vonkeman H.E., Voorn P. (1995). Preferential Loss of Preproenkephalin versus Preprotachykinin Neurons from the Striatum of Huntington’s Disease Patients. Ann. Neurol..

[76] Albin R.L., Reiner A., Anderson K.D., Dure L.S., Handelin B., Balfour R., Whetsell W.O., Penney J.B., Young A.B. (1992). Preferential Loss of Striato-external Pallidal Projection Neurons in Presymptomatic Huntington’s Disease. Ann. Neurol..

[77] Reilmann R. (2019). Parkinsonism in Huntington’s Disease. International Review of Neurobiology.

[78] Diggle C.P., Sukoff Rizzo S.J., Popiolek M., Hinttala R., Schülke J.-P., Kurian M.A., Carr I.M., Markham A.F., Bonthron D.T., Watson C. (2016). Biallelic Mutations in PDE10A Lead to Loss of Striatal PDE10A and a Hyperkinetic Movement Disorder with Onset in Infancy. Am. J. Hum. Human Genet..

[79] Langfelder P., Cantle J.P., Chatzopoulou D., Wang N., Gao F., Al-Ramahi I., Lu X.-H., Ramos E.M., El-Zein K., Zhao Y. (2016). Integrated Genomics and Proteomics Define Huntingtin CAG Length-Dependent Networks in Mice. Nat. Neurosci..

[80] Delnomdedieu M., Tan Y., Ogden A., Berger Z., Reilmann R. (2018). J06 a randomized, double-blind, placebo-controlled phase ii efficacy and safety study of the PDE10A inhibitor PF-02545920 in huntington disease (amaryllis). J. Neurolgy Neurosurg. Psychiatry.

[81] Goto S., Morigaki R., Okita S., Nagahiro S., Kaji R. (2015). Development of a highly sensitive immunohistochemical method to detect neurochemical molecules in formalin-fixed and paraffin-embedded tissues from autopsied human brains. Front. Neuroanat..

[82] Falk T., Mai D., Bensch R., Çiçek Ö., Abdulkadir A., Marrakchi Y., Böhm A., Deubner J., Jäckel Z., Seiwald K. (2019). U-Net: Deep Learning for Cell Counting, Detection, and Morphometry. Nat. Methods.

[83] Otsu N. (1979). A Threshold Selection Method from Gray-Level Histograms. IEEE Trans. Syst. Man Cybern..

[84] Franklin K.B.J., Paxinos G. (2008). The Mouse Brain in Stereotaxic Coordinates, Compact.

[85] Goldsmith P., Affinito J., McCue M., Tsai M., Roepcke S., Xie J., Gertsik L., Macek T.A. (2017). A Randomized Multiple Dose Pharmacokinetic Study of a Novel PDE10A Inhibitor TAK-063 in Subjects with Stable Schizophrenia and Japanese Subjects and Modeling of Exposure Relationships to Adverse Events. Drugs R&D.

[86] DeMartinis N., Lopez R.N., Pickering E.H., Schmidt C.J., Gertsik L., Walling D.P., Ogden A. (2019). A Proof-of-Concept Study Evaluating the Phosphodiesterase 10A Inhibitor PF-02545920 in the Adjunctive Treatment of Suboptimally Controlled Symptoms of Schizophrenia. J. Clin. Psychopharmacol..

[87] Meyer-Lindenberg A., Nielsen J., Such P., Lemming O.M., Zambori J., Buller R., Der Goltz C.V. (2022). A Double-Blind, Randomized, Placebo-Controlled Proof of Concept Study of the Efficacy and Safety of Lu AF11167 for Persistent Negative Symptoms in People with Schizophrenia. Eur. Neuropsychopharmacol..

[88] Mukai Y., Lupinacci R., Marder S., Snow-adami L., Voss T., Smith S.M., Egan M.F. (2024). Effects of PDE10A Inhibitor MK-8189 in People with an Acute Episode of Schizophrenia: A Randomized Proof-of-Concept Clinical Trial. Schizophr. Res..

[89] Zagorska A., Partyka A., Bucki A., Gawalskax A., Czopek A., Pawlowski M. (2018). Phosphodiesterase 10 Inhibitors-Novel Perspectives for Psychiatric and Neurodegenerative Drug Discovery. Curr. Med. Chem..

[90] Howes O.D., Dawkins E., Lobo M.C., Kaar S.J., Beck K. (2024). New Drug Treatments for Schizophrenia: A Review of Approaches to Target Circuit Dysfunction. Biol. Psychiatry.

