# Supplementary material for: Molecular Imbalances Between Striosome and Matrix Compartments Characterize the Pathogenesis and Pathophysiology of Huntington’s Disease Model Mouse
Source: Int J Mol Sci. 2025 Sep 3;26(17):8573. doi: 10.3390/ijms26178573 (PMC12429560; doi:10.3390/ijms26178573)
Supplement: Supplementary file 1 [file ijms-26-08573-s001.zip › ijms-3790505-supplementary.pdf]

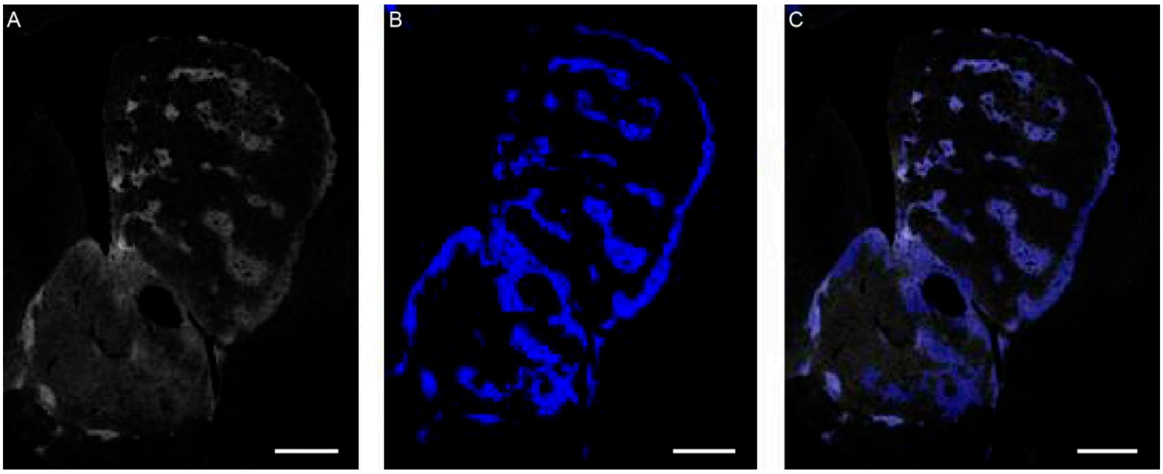

Supplementary Figure S1: Segmentation of striosomes using U-net, demonstrated with original image (A), segmented image (B), and merged image (C). Scale bars: 500 μm.

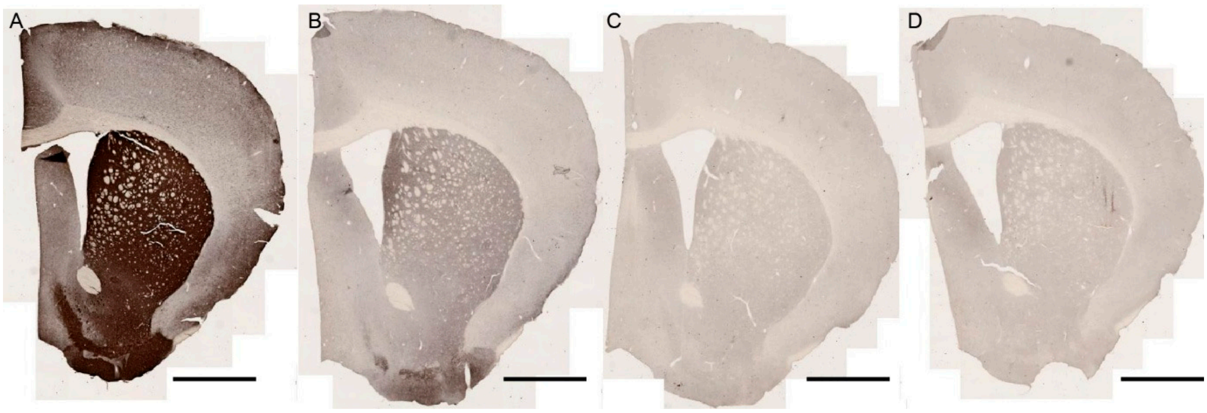

Supplementary Figure S2: Immunizing peptide assay. In comparison to positive control staining with anti-PDE10A antibody (A), the labeling of PDE10A was blocked when the primary antibody was pre-absorbed with 0.05 μg/ml (B) and 0.5 μg/ml (C) PDE10A peptide antigen. Note that the pre-absorption with high-dose peptide antigen (C) reduced PDE10A labeling equivalent to the level of negative control staining (D). Scale bar: 1 mm.

Supplementary Table S1: Performance comparison of the two models of U-net

Model	Intersection over Union (IoU)	Precision	Recall	Dice
**Vertical flipping only**	0.689788	0.749693	0.886441	0.809933
**Vertical flipping plus additional augmentation**	0.674448	0.736091	0.87602	0.797235

Supplementary Table S2: List of antibodies used in this study

Antigen	Species raised	Company	Product Code	Dilution DAB	TSA
Mu-opioid receptor	Rabbit monoclonal	Abcam, Shirley, NY	Ab134054	1:200	1:10,000
K^+ channel interacting protein 1	Mouse monoclonal	NeuroMab, Cambridge, MA	75-003	NP	1:2,000
Dopamine D1 receptor	Rat polyclonal	Sigma-Aldrich, St. Louis, MO	D2944	1:1,000	1:100,000
Dopamine D2 receptor	Rabbit polyclonal	Millipore, Billerica, MA	AB5084P	1:200	1:2,000
Adenosine 2A receptor	Goat polyclonal	Frontier Institute, Hokkaido, Japan	A2A-Go-Af700	1:5,000	1:200,000
Olfactory type G-protein α subunit	Rabbit polyclonal	Sigma-Aldrich, St. Louis, MO	SAB4501222	1:1,000	1:10,000
Phosphodiesterase10A	Rabbit polyclonal	Creative Diagnostics, Shirley, NY	DPABH-03487	1:10,000	1:100,000
Tyrosine hydroxylase	Mouse monoclonal	ImmunoStar, Hudson, WI	22941	NP	1:20,000

NP: not performed.
